# Supplementary material for: MetaRibo-Seq measures translation in microbiomes
Source: Nat Commun. 2020 Jun 29;11:3268. doi: 10.1038/s41467-020-17081-z (PMC7324362; doi:10.1038/s41467-020-17081-z)
Supplement: Supplementary file 10 — Supplementary Data 7 [file 41467_2020_17081_MOESM10_ESM.zip › File2/Confidence_High_Taxonomy/420871_out.krona.html]

Javascript must be enabled to view this page.

members
magnitude
magnitudeUnassigned
count
unassigned
taxon
rank

420871\_out

78

superkingdom
78
2

976
78
phylum

class
200643
78

order
171549
78

78
815
family

genus
78
816

species

SRS046712\_contig\_number\_164SRS147272\_contig\_number\_42
2
2291999

species
817
75

SRS011084\_contig\_number\_35484SRS012902\_contig\_number\_1283SRS013158\_contig\_number\_4385SRS013215\_contig\_number\_1502SRS014613\_contig\_number\_contig-100\_716.112097SRS014736\_contig\_number\_698SRS015578\_contig\_number\_20826SRS015854\_contig\_number\_23366SRS015890\_contig\_number\_6034SRS015960\_contig\_number\_3208SRS016095\_contig\_number\_29227SRS016132\_contig\_number\_520SRS017247\_contig\_number\_contig-100\_876.53201SRS017521\_contig\_number\_50587SRS017622\_contig\_number\_331SRS018313\_contig\_number\_5794SRS018656\_contig\_number\_contig-100\_88.76511SRS018817\_contig\_number\_23625SRS018984\_contig\_number\_5154SRS019267\_contig\_number\_8202SRS019601\_contig\_number\_29279SRS020869\_contig\_number\_contig-100\_758.217801SRS021484\_contig\_number\_7019SRS023346\_contig\_number\_3983SRS023583\_contig\_number\_11084SRS023829\_contig\_number\_352SRS023971\_contig\_number\_4127SRS024075\_contig\_number\_403SRS024132\_contig\_number\_375SRS024549\_contig\_number\_4479SRS046502\_contig\_number\_10831SRS053214\_contig\_number\_3044SRS053573\_contig\_number\_3280SRS055966\_contig\_number\_455SRS056259\_contig\_number\_15266SRS057717\_contig\_number\_2209SRS058723\_contig\_number\_contig-100\_22.60906SRS063127\_contig\_number\_2319SRS063190\_contig\_number\_14746SRS063985\_contig\_number\_29238SRS064276\_contig\_number\_35280SRS074964\_contig\_number\_6697SRS075398\_contig\_number\_3214SRS075878\_contig\_number\_16499SRS076804\_contig\_number\_10178SRS077194\_contig\_number\_26420SRS077335\_contig\_number\_2549SRS077589\_contig\_number\_6710SRS077730\_contig\_number\_12990SRS097920\_contig\_number\_25048SRS098644\_contig\_number\_41110SRS098655\_contig\_number\_4886SRS098881\_contig\_number\_1308SRS1041039\_contig\_number\_7080SRS1041145\_contig\_number\_25257SRS104311\_contig\_number\_37601SRS104485\_contig\_number\_1300SRS104636\_contig\_number\_30943SRS1054928\_contig\_number\_6023SRS1055043\_contig\_number\_23972SRS1055067\_contig\_number\_9792SRS1055099\_contig\_number\_10557SRS140492\_contig\_number\_25441SRS142599\_contig\_number\_30202SRS143342\_contig\_number\_2612SRS143417\_contig\_number\_7082SRS144135\_contig\_number\_35869SRS144362\_contig\_number\_13442SRS146812\_contig\_number\_29669SRS147022\_contig\_number\_1507SRS147139\_contig\_number\_9519SRS147377\_contig\_number\_650SRS148159\_contig\_number\_57423SRS149075\_contig\_number\_12638SRS893342\_contig\_number\_711

1

SRS049402\_contig\_number\_17014
28116
species
